# Quantification of the effects of an alpha-2 adrenergic agonist on reflex properties in spinal cord injury using a system identification technique

**DOI:** 10.1186/1743-0003-7-29

**Published:** 2010-06-23

**Authors:** Mehdi M Mirbagheri, David Chen, W Zev Rymer

**Affiliations:** 1 Department of Physical Medicine and Rehabilitation, Northwestern University, Chicago, USA; 2 Sensory Motor Performance Program, Rehabilitation Institute of Chicago, Chicago, USA

## Abstract

**Background:**

Despite numerous investigations, the impact of tizanidine, an anti-spastic medication, on changes in reflex and muscle mechanical properties in spasticity remains unclear. This study was designed to help us understand the mechanisms of action of tizanidine on spasticity in spinal cord injured subjects with incomplete injury, by quantifying the effects of a single dose of tizanidine on ankle muscle intrinsic and reflex components.

**Methods:**

A series of perturbations was applied to the spastic ankle joint of twenty-one spinal cord injured subjects, and the resulting torques were recorded. A parallel-cascade system identification method was used to separate intrinsic and reflex torques, and to identify the contribution of these components to dynamic ankle stiffness at different ankle positions, while subjects remained relaxed.

**Results:**

Following administration of a single oral dose of Tizanidine, stretch evoked joint torque at the ankle decreased significantly (p < 0.001) The peak-torque was reduced between 15% and 60% among the spinal cord injured subjects, and the average reduction was 25%. Using systems identification techniques, we found that this reduced torque could be attributed largely to a reduced reflex response, without measurable change in the muscle contribution. Reflex stiffness decreased significantly across a range of joint angles (p < 0.001) after using tizanidine. In contrast, there were no significant changes in intrinsic muscle stiffness after the administration of tizanidine.

**Conclusions:**

Our findings demonstrate that tizanidine acts to reduce reflex mechanical responses substantially, without inducing comparable changes in intrinsic muscle properties in individuals with spinal cord injury. Thus, the pre-post difference in joint mechanical properties can be attributed to reflex changes alone. From a practical standpoint, use of a single "test" dose of Tizanidine may help clinicians decide whether the drug can helpful in controlling symptoms in particular subjects.

## Introduction

Spasticity can disrupt activities of daily life [1,2], and has substantial physical, emotional and social costs [2]. Appropriate treatment of spasticity would therefore have many benefits. Since spasticity is manifested as a mechanical abnormality, to evaluate the therapeutic effects of treatments, there is a need to quantify these effects on neuromuscular mechanics. The current study was designed to determine the impact of an important anti-spasticity medication, tizanidine, by quantifying the effect of single dose tizanidine on reflex and intrinsic components of muscle response to stretch in individuals with spinal cord injury. The value of such assessments could also be to enhance our capacity to predict which patients will respond well to the agent in the course of long-term treatment.

"Hypertonia" is one of the primary clinical features associated with spinal cord injury [2,3]. Hypertonia, defined as an abnormal increase in muscle tone [4], is a defining feature of spasticity and has both diagnostic and therapeutic significance [5]. Many antispastic drugs such as baclofen, diazepam, and clonidine decrease hypertonia [6-12]. Most, however, have significant adverse effects [7,11,13]. The present study focuses on tizanidine, an α_2 _noradrenergic (NE) agonist, which is incompletely studied, but is thought to act to depress dorsal horn interneuron excitability [14]. It has been shown to be at least as effective as other antispastic agents [6,15-17] and often better tolerated [13,15,17,18], with mild side effects such as sedation [10,12,13], muscle weakness [10-12], and decreased vasomotor responses [19]. Further, experimental evidence in spinalized cats demonstrate marked improvements in locomotor capacity following intrathecal delivery of α_2 _NE agonists [20].

Tizanidine is an α_2_-adrenergic agonist and presumably acts on presynaptic terminals and on interneurons [21,22] within the spinal cord to restore noradrenergic inhibition, mostly on polysynaptic pathways, that may promote spasticity [19,23]. Thus, the spectrum of activity for tizanidine is broad, making it likely that tizanidine affects different symptoms of spasticity such as hypertonia, flexor reflexes, spasms and clonus.

A number of studies have examined the therapeutic effects of tizanidine [7-9,11,13,15,24]. Decreases in spastic symptoms such as hypertonia, spasms and clonus have been reported in spastic spinal cord injured patients [21,24-28]. These findings were based on clinical evaluations of spasticity such as Ashworth scale and on the pendulum test [17,24]. These approaches used different criteria and were inherently subjective and qualitative in nature.

More importantly, although hypertonia arises broadly from changes in the mechanical properties of passive tissues, muscles, and from abnormal reflexes [29-31], these qualitative approaches were not able to quantify the effects of tizanidine since they did not routinely differentiate intrinsic and reflex contributions to spastic hypertonia. Thus, the mechanisms of action of tizanidine on neuromuscular properties in spinal cord injured subjects remain unknown. The objective of this study was therefore to address this deficit by quantifying the effect of single dose tizanidine on reflex and intrinsic contributions to overall ankle joint stiffness in spastic spinal cord injured subjects.

In earlier publications, we described a system identification method to characterize joint dynamic stiffness and to separate it muscular (intrinsic) and reflex contributions to overall stiffness analytically [30,32]. We showed that both reflex and intrinsic stiffness were abnormally high in spastic spinal cord injured subjects and that consequently overall stiffness increased significantly [30]. In view of clinical reports that tizanidine reduces spasticity, we hypothesized that these mechanical abnormalities, particularly reflex stiffness, would decrease following administration of tizanidine.

## Methods

### Subjects

Forty individuals with incomplete spinal cord injured (37.6 ± 13.2 years) participated in this study. All subjects had chronic spinal cord injury of between 2 and 18 years (8.5 ± 4.7 years) duration, with different degrees of spasticity. Twenty subjects received tizanidine and repeated joint perturbations including tests before and after tizanidine administration. Twenty served as controls, receiving no tizanidine and a more limited set of joint perturbations. Two subjects in the tizanidine group were unable to complete data collection because of their time constraints, and their data are not included here.

Patients had sustained a traumatic, motor incomplete non-progressive spinal cord injury, with an American Spinal Injury Association (ASIA) impairment scale classification of C or D indicating motor incomplete lesions. In addition, the neurological level of injury was above T10, spasticity was present in the leg, and the study was initiated a minimum of 1 year post-injury.

Subjects gave informed consent to the experimental procedures, which had been approved by Northwestern University Institutional Review Board.

### Clinical assessment

All spinal cord injured subjects were evaluated clinically using the modified 6-point Ashworth scale to assess the severity of spasticity [33,34]. (The modified Ashworth scale is a standard conventional clinical measure of spasticity.) Subjects' modified Ashworth scale scores varied between 1 and 4.

In spinal cord injured subjects with incomplete motor function loss, the sides of the body are often affected differently, so both sides were assessed in this study. The side with the highest modified Ashworth scale and lowest isometric maximum ankle plantarflexion torque, which was always on the same side, was studied.

### Apparatus

The joint stretching motor device operated as a position control servo driving ankle position to follow a command input. Subjects were seated and secured in an adjustable chair with the ankle strapped to the footrest and the thigh and trunk strapped to the chair. The seat and footrest were adjusted to align the ankle axis of rotation with the axis of the force sensor and the motor shaft. An oscilloscope mounted in front of the subject displayed a target signal and provided visual feedback of low-pass filtered joint torque.

Movements in the plantarflexion direction were taken as negative and those in the dorsiflexion direction as positive. A 90° angle of the ankle joint, measured between the lateral malleolus and the foot plat, was considered to be the neutral position (NP) and defined as zero.

Electromyograms (EMGs) were recorded from tibialis anterior and lateral gastrocnemius for the ankle joint using bipolar surface electrodes (Delsys, Inc. Boston, MA). Position, torque, and EMGs were filtered at 230 Hz to prevent aliasing, and sampled at 1 kHz by a 16 bit A/D.

### Experimental Procedures

#### Administration of tizanidine

We provided a single dose of 2 mg, a relatively small dose. This dose level usually shows effects, but does not cause major side effects. Thus, this can be tolerated by vast majority of patients. Subjects were tested before and after the administration of tizanidine. Tizanidine effects begin within 30 minutes, and last for up to 4 hours, so we began recording 60 minutes after drug administration..

#### Isometric maximum voluntary contraction

Isometric maximum voluntary conractions were determined by having subjects contract the ankle muscles maximally toward plantarflexion and dorsiflexion directions at neutral position (90°); torque and EMG were sampled for 5 sec. Measurements were repeated at least twice and the best measure was considered as maximum voluntary contraction.

#### Range of motion

Range of motion was determined with the subject's ankle attached to the motor. The ankle range of motion was recorded using the goniometer built onto the motor, but under passive conditions, to guarantee safety Mean amplitude was estimated by slowly moving the joint until the examiner perceived rapidly increasing resistance or the subject reported discomfort. Measurements were done 3 times.

#### Stretch trials

To evaluate the peak-torque responses after tizanidine, a series of 5 large, rapid stretch and hold dorsiflexion perturbations was applied to the ankle joint with displacement amplitude of 30°, stretch velocity of 200 deg/s, and hold time of approximately 2.4 s. This movement velocity is routinely sufficient to elicit stretch reflex responses. Stretch trials were applied with the joint in 20° plantarflexion and the torque and EMG responses were ensemble-averaged.

#### Pseudorandom binary sequence trials

To identify overall stiffness properties and to separate the reflex and intrinsic components, we used pseudorandom binary sequence inputs with amplitude of 2° and a switching interval of 150 ms. These perturbations were applied to the ankle of drug recipients and to controls for a period of 30 s at each joint position. Trials were conducted at multiple different joint positions from full plantar flexion to maximum tolerable dorsiflexion at 5° intervals.

These perturbations are appropriate to characterize the joint dynamic stiffness and to separate its neural and muscular components [32] and they are well tolerated [29-31]. The random nature of these perturbations makes it difficult for the subject to generate a systematic voluntary response. To control for muscle activation changes during the perturbation, we monitored EMG's of both ankle flexors and extensor. Subjects were asked to remain relaxed and not to react voluntarily to the perturbation sequence. If there was evidence of a voluntary reaction, the experimental trials were repeated. Following completion of each trial, the torque and EMG signals were examined for non-stationarities or co-activation of other muscles. If there was evidence of either, the data were discarded and the trial was repeated.

#### Control Pulse Trials

Pulse trials were applied before and after pseudorandom binary sequence trials to control for changes in the subject's state, as quantified by the overall level of excitability of motoneurons. Five small pulses with an amplitude of 2° and pulse-width of 40 ms were applied to the ankle; EMGs from tibialis anterior and gastrocnemius and ankle torque were recorded and ensemble-averaged. The amplitudes of the reflex responses were used as an empirical measure of stretch reflex excitability. Changes in response amplitudes greater than 10% were taken as evidence of a change in the subject's state, due to fatigue or other factors, and data for the trial were discarded. This occurred very rarely; in most experiments no trials were discarded.

### Analysis procedures

#### Parallel cascade system identification model

We used a parallel cascade system identification technique to separate reflex and intrinsic contributions to ankle dynamic stiffness. This technique, described in detail in earlier publications [32,35].

The intrinsic stiffness was estimated in terms of a linear Impulse Response Function (IRF), which is a curve relating position and torque. The IRF characterizes the behavior of the system over its entire range of frequencies. The intrinsic IRF was convolved with the experimental input to predict the intrinsic torque.

The reflex pathway was modeled as a differentiator in series with a delay, a half-wave rectifier (indicating the direction of stretch), and a dynamic linear element. Reflex stiffness was estimated by determining the IRF between half-waved rectified velocity as the input and reflex torque (the difference between the net joint torque and the predicted intrinsic torque) as the output. The reflex IRF was convolved with the half-waved velocity, as input, to predict the reflex torque.

IRFs were assessed in terms of the percentage of the output (torque) variance accounted for.

Intrinsic and reflex stiffness gains were calculated by fitting linear models to their IRF curves [32].

#### Statistical analysis

We used two-way ANOVA analyses to test for significant main effects due to tizanidine, joint positions, or their interactions. The results can tell us if there were significant differences due to main effects and/or their interactions. Tukey post-hoc comparisons were performed to find at which positions the differences between pre- and post-tizanidine were significant.

Standard t-tests procedures were used to test for significant changes in peak torque and maximum voluntary contractions due to tizanidine.

## Results

### tizanidine effects on stretch-induced torque and maximum voluntary contraction

**Figure 1:**
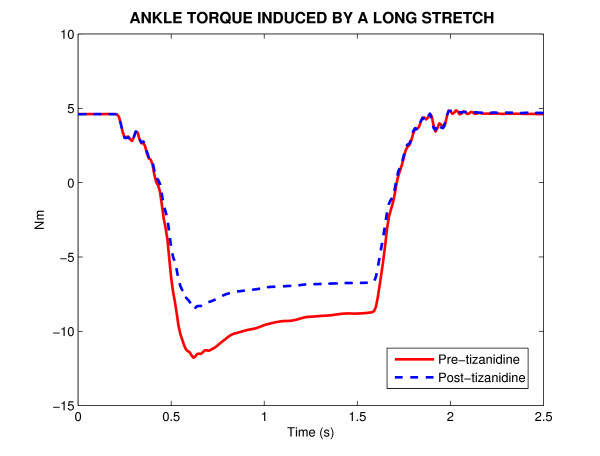
**The ankle torque induced by a long-stretch trial with displacement amplitude of 30° and duration of 2.5 s around neutral position (90°) before, (Pre) and after, (Post) tizanidine administration of a single dose of the drug in a spinal cord injured subject**. Pre-tizanidine (solid-lines) and post-tizanidine (dashed-lines).

Figure 1 shows a typical position stretch trial with displacement amplitude of 30° and duration of 2.5 s, which stretched the ankle joint around the neutral position. The ankle torque induced by this stretch is shown before, (Pre) and after, (Post) tizanidine administration of a single dose of the drug. It is to be noted that peak-torque was reduced substantially (35%) after taking this single dose of tizanidine.

**Figure 2:**
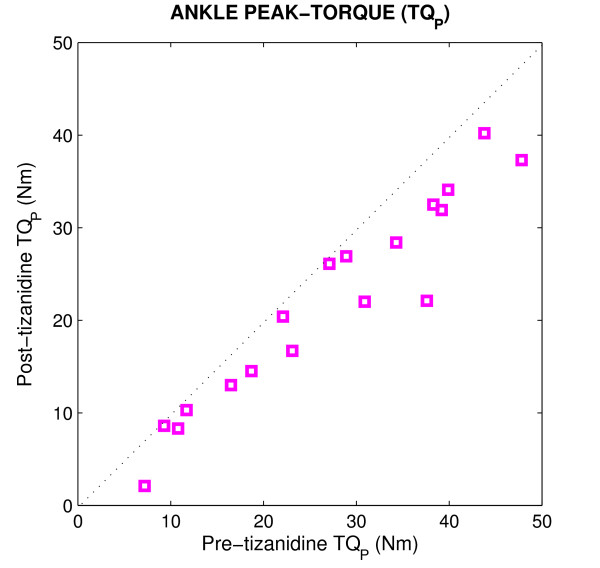
**Post-tizanidine peak-torque (TQ_P_) values plotted against pre-tizanidine TQ_P _values for all subjects**.

Figure 2 shows the peak-torque response of the spastic ankle, comparing responses, Post vs Pre for all subjects. The dotted line at 45 degrees (the unity line) in each panel indicates what would be expected if there were no change due to the medication. Points below the line indicate decreases following the administration of tizanidine, while points above the line indicate abnormal increases. The peak-torque values for all subjects were located well below the diagonal line, indicating that peak-torque decreased significantly (p < 0.001) post-tizanidine. The peak-torque was reduced between 15% and 60% among the spinal cord injured subjects, and the average of changes was 25%.

In contrast to the peak torque, there was no significant difference in maximum voluntary contractions between Pre and Post tizanidine administration.

### tizanidine effects on reflex and intrinsic stiffness

The torque response has two distinct components (Figure 1); a component correlated with ankle position and its derivatives, beginning with no delay, and attributable to intrinsic mechanics, and a transient component associated with dorsiflexion displacements only, likely representing the contribution of stretch reflex mechanisms in plantar-flexor muscles. To identify the intrinsic and reflex contributions to overall joint torque net and to estimate their mechanical properties, we used the parallel cascade system identification technique.

**Figure 3:**
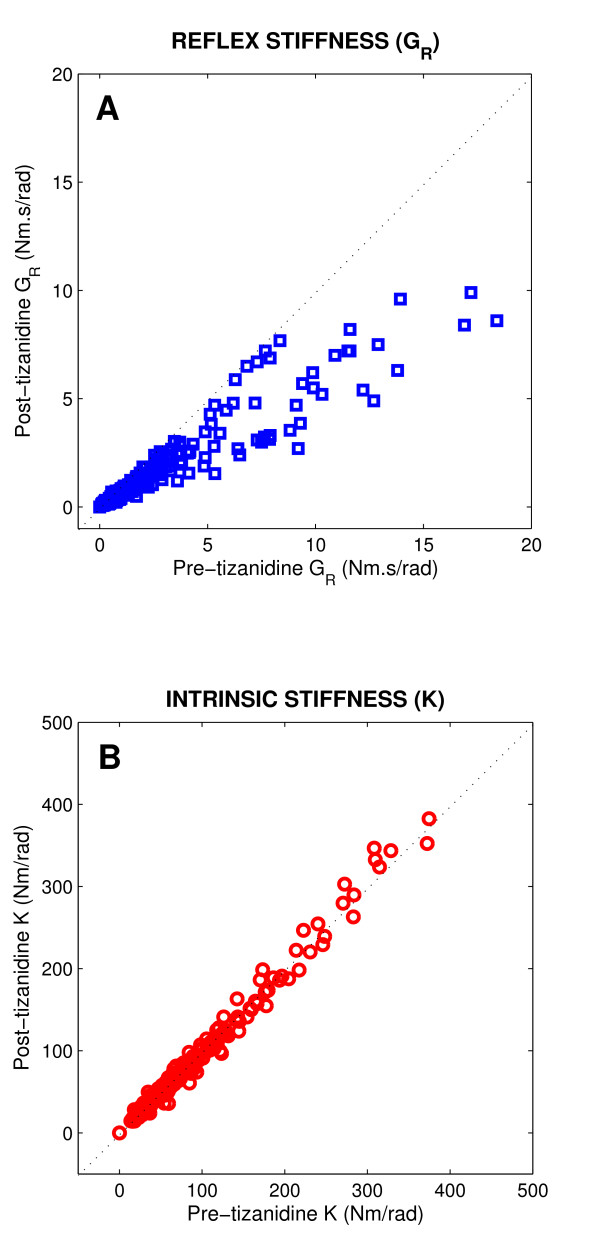
**Post-tizanidine stiffness values plotted against pre-tizanidine values for all subjects**. **A **Reflex stiffness gain (*G*_*R*_), **B **Intrinsic stiffness gain (*K*).

Figure 3 shows reflex stiffness gain (*G*_*R*_, panel A) and intrinsic stiffness gain (*K*, panel B) of the spastic ankle post- versus pre-tizanidine for all spinal cord injured subjects, and for all positions. *G*_*R *_for all subjects were well located below the diagonal line, indicating that *G*_*R *_was significantly lower Post as compared with Pre drug administration (p < 0.001). In contrast, the points for the *K *were mostly clustered around the unity line, and did not show significant differences between post- and pre-tizanidine. These results demonstrate that the significant decrease observed in peak-torque responses after tizanidine (Figure 2) was due primarily to a reduction in reflex torque response.

### tizanidine effects on modulation of reflex and intrinsic stiffness with joint angle

Our previous studies demonstrated that *G*_*R *_and *K *were strongly dependent on joint position. Consequently, we decided to examine the position dependence of the changes with tizanidine.

**Figure 4:**
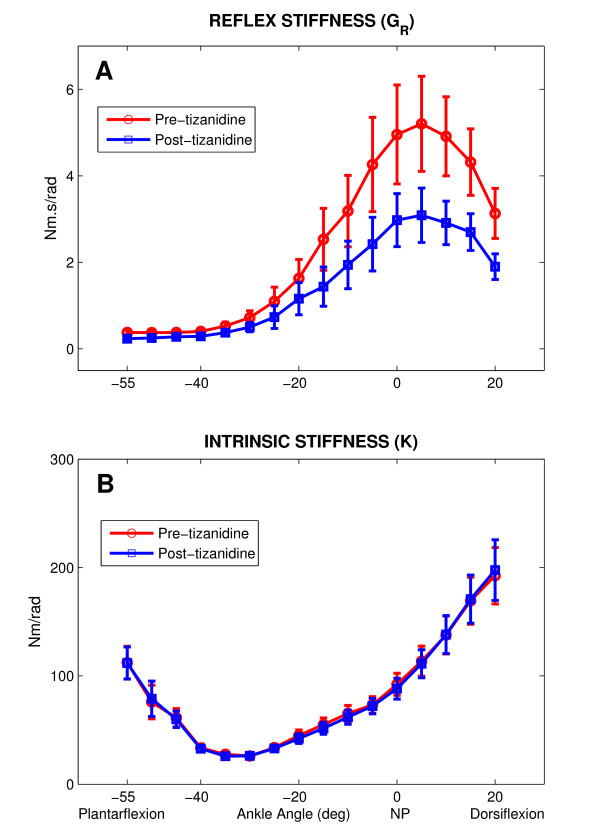
**Reflex and intrinsic stiffness gain as a function of ankle angle (Group averages)**. **A **Reflex stiffness gain (*G*_*R*_), **B **Intrinsic stiffness gain (*K*). Pre-tizanidine (circles) and post-tizanidine (squares). Error bars indicate ±1 standard errors. NP: Neutral Position (90°).

Figure 4 shows group average results for *G*_*R *_(panel A) and *K *(panel B) as a function of ankle position for both Pre and Post treatment groups. There was a significant effect due to position on both the Pre and Post groups (p < 0.001). The differences increased when ankle was stretched toward dorsiflexion, reached its maximum at the first 5° dorsiflexion, and declined sharply with further dorsiflexion. Tukey post-hoc analysis confirmed that (*G*_*R*_) was significantly lower in Post vs Pre treatment group, from mid-plantarflexion (20° plantarflexion) to full-dorsiflexion (20° dorsiflexion) (p < 0.001).

The group position-dependent behavior was consistent but the inter-subject variability was high from mid-plantarflexion to full-dorsiflexion as demonstrated by the large standard error bars associated with the means.

In contrast to *G*_*R*_, there was no significant difference in *K *between Pre and Post treatment groups (Fig. 4B). Furthermore, the behavior of *K *for both Pre and Post group with changes in ankle joint angle was very consistent, as demonstrated by the narrow standard error bars.

To characterize the amplitude of these changes for each spinal cord injured subject, we studied tizanidine effects for all positions over the range of motion. Thus, we first computed the percentage of changes caused by tizanidine at each position for *G*_*R *_and then averaged them for each spinal cord injured subject.

**Figure 5:**
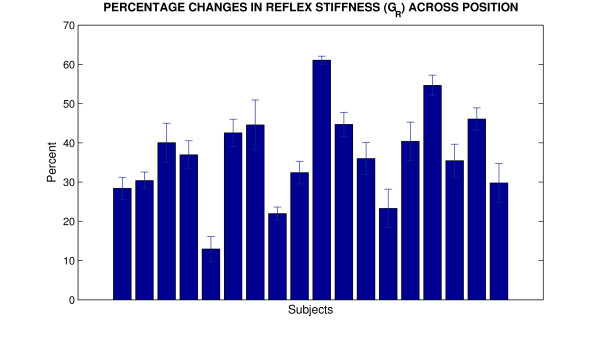
**Percentage change of tizanidine effects on reflex stiffness gain (*G*_*R*_) for each SCI subject (Position averages)**. Error bars indicate ±1 standard errors.

Figure 5 shows the tizanidine effects on *G*_*R *_for all spinal cord injured individuals. *G*_*R *_decreased between 14% and 57% for all subjects with the average of 38%. Interestingly, the inter-subject variability was small (11%) as changes were more than 30% in most subjects (more than 75% of subjects), indicating consistent impact of the single dose on reflex mechanical responses. The small standard deviation error bars show that the changes were independent of position.

### Control ***Group***

We did not find a significant change in control subjects' state over the course of each experiment, as evidenced by the absence of changes exceeding 10% in reflex torque. Because of this, we limited the pseudorandom binary sequence trials only at the neutral position in the control group. We found no significant changes in *G*_*R *_and *K *values between this final and initial measure. This indicates that the changes observed following tizanidine were attributable solely to the actions of the drug, and not to time or history dependent changes in reflex response.

## Discussion

The focus of this study was to quantify the effects of single dose of tizanidine on the neuromuscular abnormalities associated with spasticity, with particular emphasis on the relative contributions of changes in reflexes versus changes in intrinsic neuromuscular properties.

Prior studies on tizanidine have focused mostly on clinical measurements of spasticity or weakness. Many of these studies, have investigated the effects of tizanidine on spasticity using the Ashworth scale and the pendulum test [17,24]. Others have used EMG responses to evaluate the effects of the drug on reflex behavior [26,36,37]. However, a conclusive link between the clinical measures and reflex mechanical properties remains elusive [38]. Furthermore, clear connections between reflex EMG and muscular hypertonia have also not yet been established [30]. This is likely due to the complex, nonlinear interactions between muscles' contractile properties and activation dynamics which make it difficult to predict torque on the basis of EMG alone [32,39]. Our study sought to assess the impact of oral tizanidine on reflex and mechanical properties of spastic muscle, using a system identification technique. In our earlier study, we have used this technique to characterize the neuromuscular abnormalities associated with spasticity and to quantify the effect of long-term use of FES-assisted walking on neuromuscular properties in subjects with spinal cord injury [40]. In parallel, we determine the magnitude of each component's contribution to the physical sign of muscular hypertonia, a key clinical feature of spasticity.

Our study findings are that the actions of tizanidine, an α_2 _adrenergic agonist on stretch reflexes in spastic subjects are substantial, and that they take effect quickly, and at a relatively small dose. A single 2 mg dose of tizanidine was routinely effective, reducing joint torque substantially within 60 minutes, by a magnitude of 15% to 60%.

Second, though perhaps to be anticipated, the study affirms that the effects of the agent on limb spasticity are mediated by reflex changes, rather than by direct changes in muscle contractility. Although this is the accepted view of tizanidine actions, there have been reports of loss of voluntary muscle strength [9,10,12], which have triggered some doubt on this issue. In our hands, the maximum voluntary contraction's were unmodified, yet changes in reflex contributions to joint torque were substantial, rapid and consistent. Reports of weakness are particularly prevalent during gait, where a stiff/spastic leg may serve as a strut, helping to support body weight. So the apparent loss of strength in this situation may be linked to reductions in spasticity of major leg muscles.

It is likely that the actions of the agent are mediated, at least in part, at interneuronal sites [21,22], and not just at motoneuron synapses. Tizanidine actions are believed to be mediated via the restoration of inhibitory suppression of the group II spinal interneurons by the α-adrenergic agent [14]. These interneurons are excitatory to spinal motor neurons, and may underlie the enhanced depolarization that accompanies spastic hyperreflexia [14,41].

Finally, the findings of our systems identification methods affirm that changes in joint torque following a single dose of tizanidine are attributable almost entirely to changes in stretch reflex contributions, without changes in the intrinsic muscle contribution to net joint torque. Although these (reflex) changes conform with the known physiology/pharmacology of tizanidine [19,23], it may also emerge that the response to low dose tizanidine proves to be a strong predictor of clinical/therapeutic efficacy, in that clear reductions in reflex torque for a small dose may signify the likelihood of strong therapeutic effects of the drug.

## Competing interests

The authors declare that they have no competing interests.

## Authors' contributions

MMM designed the study, supervised data collection and analysis, and participated in interpreting and writing the manuscript. DC referred the patients and participated in interpreting data, and WZR participated in interpreting data and writing the manuscript. All authors read and approved the final manuscript.
